# Associations of Area-Level and Parental Individual-Level Social Deprivation with Glycemic Control over Time among Children with Type 1 Diabetes in France: A Longitudinal Cohort Study

**DOI:** 10.1155/2024/9634867

**Published:** 2024-05-20

**Authors:** Isaline Morard, Pascal Barat, Marion Bailhache

**Affiliations:** ^1^Pediatric Endocrinology and Diabetes Unit, Children's Hospital, Bordeaux University Hospital, Place Amélie Raba-Léon, 33076, Bordeaux Cedex, France; ^2^Pediatric Emergency Unit, Children's Hospital, Bordeaux University Hospital, Place Amélie Raba-Léon, 33076, Bordeaux Cedex, France; ^3^ISPED, INSERM Unit U1219 Bordeaux Population Health, Bordeaux University, 146 rue Léo Saignat, Bordeaux Cedex 33076, France

## Abstract

**Background:**

Poor glycemic control in patients with type 1 diabetes (T1D) is associated with greater social deprivation. However, the evidence is inconsistent in terms of the type of social deprivation (individual-level or area-level) and whether glycemic control changes over time. Here, we investigated the impacts of individual-level and area-level social deprivation on the glycated hemoglobin (HbA1c) trajectory from the time of T1D diagnosis.

**Materials and Methods:**

We retrospectively analyzed a cohort of children who were diagnosed with T1D between 2017 and 2020 at Bordeaux University Hospital. Social deprivation was assessed using both parental individual indicator (EPICES score) and ecological indicator (European Deprivation Index (EDI) score). Piecewise linear mixed-effects models were used to estimate the effects of social deprivation on HbA1c trajectory.

**Results:**

We included 168 patients. The most-deprived group included 29% and 22% of all patients, as revealed by the respective EPICES and EDI scores. The two indicators were poorly correlated. The short-term decrease in HbA1c level tended to be smaller in the most-deprived patients over the first 4 months after diagnosis than in other patients (slope difference of 2.68% per year compared with the slope among the least-deprived patients, *P* = 0.056). The long-term trajectory was influenced by area-level deprivation (EDI score); the least-deprived patients (quintile 1) exhibited more stable mean HbA1c levels.

**Conclusions:**

Social deprivation may partially explain poor glycemic control in some patients; both short-term individual deprivation and long-term area-level deprivation may be involved. Further research is needed to determine how to integrate this information into a therapeutic strategy.

## 1. Introduction

Optimal glycemic control is necessary to reduce the risk of diabetes complications and improve the quality of life in affected patients. Despite the introduction of new technologies for the management of type 1 diabetes (T1D), many children and adolescents do not meet their glycated hemoglobin (HbA1c) targets; thus, they are at risk of developing serious complications [1]. There is a need to understand why these targets are unmet and then remove the obstacles. Social inequalities affect the progression of many chronic diseases that begin in early life; such inequalities may influence metabolic control in young patients with T1D. Several international studies have shown that poor glycemic control is associated with greater social deprivation at either the individual level or the ecological level [2–10]. However, these were mostly cross-sectional studies that did not consider changes in HbA1c levels over time. Only a few studies have used both individual and ecological indicators to assess social deprivation. In France, there are limited data regarding social inequalities in childhood, as well as the effects of inequalities on T1D; further research is needed to guide new management strategies [11, 12]. The primary objective of this study was to determine the association between the HbA1c trajectory and social deprivation in children who were newly diagnosed with T1D. We used two composite indicators of deprivation: the individual indicator EPICES (“Evaluation de la Précarité et des Inégalités de Santé dans les Centres d'Examens de Santé,” Evaluation of the Deprivation and Inequalities of Health in Healthcare Centers) and the ecological indicator European Deprivation Index (EDI). We also evaluated the relationship between these indicators.

## 2. Materials and Methods

This retrospective longitudinal cohort study was conducted from 2017 to 2022 in a tertiary university hospital (Hôpital des Enfants) in Pellegrin, Bordeaux, France.

### 2.1. Study Population

We included patients aged <18 years who were diagnosed with T1D at Bordeaux University Hospital between January 1, 2017, and December 31, 2020. A minimum of one postdiagnosis follow-up visit to the same hospital was required. If the diabetes was not T1D (i.e., it was type 2, monogenic, or neonatal diabetes) or the T1D diagnosis was uncertain, the patient was excluded. Patients residing outside France and patients lacking a permanent address were also excluded.

### 2.2. Study Measures

#### 2.2.1. Primary Outcome

The outcome of interest was the evolution of glycemic control over time. We used the percentage HbA1c level, which reflects the mean glycemic level over the previous 3 months [13]. HbA1c levels were measured in venous or capillary blood using standard methods. HbA1c measurements were conducted at diagnosis and during all follow-up visits. HbA1c was not measured at the same time for each patient because there was no standardized protocol for follow-up (retrospective study). Repeat measurements were grouped at 4-month intervals because this corresponded to the frequency of follow-up of diabetic patients observed in practice and allowed a sufficient number of patients to be included in each interval. If the same patient had more than one visit in a given time interval, the mean HbA1c was used.

#### 2.2.2. Exposures

The principal exposure factor was social deprivation, assessed using parental individual-level and area-level (ecological) indicators. Parental individual social deprivation was assessed by deriving the EPICES score, which was computed using answers to 11 binary questions that explore >90% of the individual social gradient. The score ranges from 0 (minimum deprivation) to 100 (maximum deprivation) (Appendix S1). A value of 30.17 (baseline quintile 4 level) is regarded as the threshold of instability [14]. The EPICES score exhibited good reliability in terms of measuring individual deprivation, when compared with the reference indicators of Townsend and Carstairs [15]. The EPICES score has been used by the Division of Pediatric Diabetes at Bordeaux University Hospital since 2016. The questionnaire was routinely administered in a face-to-face interview with one or both parents during the hospitalization of the child after diagnosis with diabetes or during a follow-up visit or a new hospitalization. Because some items may have been influenced by the severe acute respiratory syndrome coronavirus 2 (SARS-CoV-2) pandemic, parents were asked to answer based on their pre-pandemic experiences. If the parents were separated, the score of the parent with primary custody was considered. If custody alternated equally, the best (lowest) score was used. Area-level social deprivation was measured using the EDI. This composite ecological score is based on a selection of variables that best reflect the individual dimensions of deprivation [16]. We used the 2015 French version of the EDI, calculated as follows:

(1) $$\begin{array}{c} \text{F-EDI }2015=0.50\;x\%\text{ no access to a car}+0.84\;x\%\text{ non-owner}+0.44\;x\%\text{ overcrowding}+0.64\;x\%\text{ low level of education}+0.97\;x\%\text{ unskilled worker}+0.73\;x\%\text{ foreign nationality}+1.11\;x\%\text{ single-parent household}+0.25\;x\%\text{ households of two or more persons}+0.97\;x\%\text{ unemployment}+0.39\;x\%\text{ not married}. \end{array}$$

The geographic unit used was the “Ilots Regroupés pour l'Information Statistique” (IRIS), which is the smallest geographical census unit in France [17]. The continuous EDI score is divided into national quintiles and most often used in this manner, such that a higher quintile indicates a more deprived area. An IRIS was assigned to each patient based on their residential address. The corresponding IRIS was obtained from the child's address. Then, the corresponding EDI value was provided by the Aapriss platform.

#### 2.2.3. Covariates

**Figure 1:**
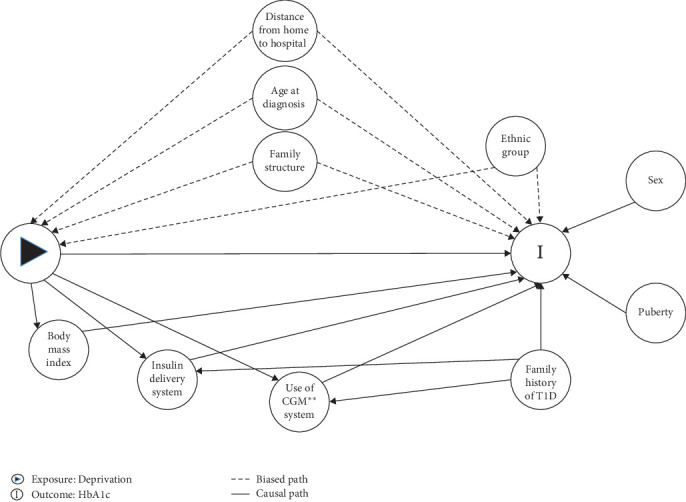
Directed acyclic graph of the relationship between social deprivation and glycemic control. *⁣*^*∗∗*^CGM, continuous glucose monitoring.

Potential confounders of the relationship between deprivation and changes in the HbA1c level over time were identified by collecting expert opinions regarding T1D through a literature review and using the directed acyclic graph shown in Figure 1. Adjusted covariates were age at diagnosis, type of family structure, and distance from home to Bordeaux University Hospital (km).

### 2.3. Data Source

Data were collected at the time of T1D diagnosis; all follow-up visits were retrospectively extracted from electronic patient records and anonymized. The collected data included sociodemographic information, family characteristics, clinical and biological characteristics, and diabetes features and management.

### 2.4. Statistical Analysis

Continuous variables are presented as medians (interquartile ranges), and categorical variables are presented as frequencies. Associations between the two indicators were investigated by deriving the Spearman correlation (for continuous scores) and Cohen's weighted kappa (for quintiles). Multiple imputation was performed using the Multivariate Imputation by Chained Equations (MICE) method; this was sufficient to manage missing values of exposures and covariates. Ten tables, each of 10 iterations, were constructed. Linear mixed-effects models with random intercepts and slopes were used to study the effects of social deprivation on the HbA1c level at T1D diagnosis and over time (% per year). Mixed models aid longitudinal data analysis because they manage correlations among different data points regarding the same individual [18]. To manage the nonlinear evolution of the HbA1c level over time, we used piecewise mixed models with cutoff times set at 4 months postdiagnosis. The indicators of social deprivation served as categorical variables in separate models; these were EPICES scores < or ≥ 30.17 (Model 1) and the EDI quintiles (Model 2). Interaction terms between time and the indicators of social deprivation were included to assess changes in the effects of the indicators over time. All models were adjusted for confounders. We assessed the underlying mixed model assumptions: normality and the homoscedasticity of residuals. Two-sided tests were performed with the statistical significance threshold set at *P*  < 0.05. All statistical analyses were performed using R (version 4.0.3) and R Studio (version 1.3.1093).

## 3. Results

**Figure 2:**
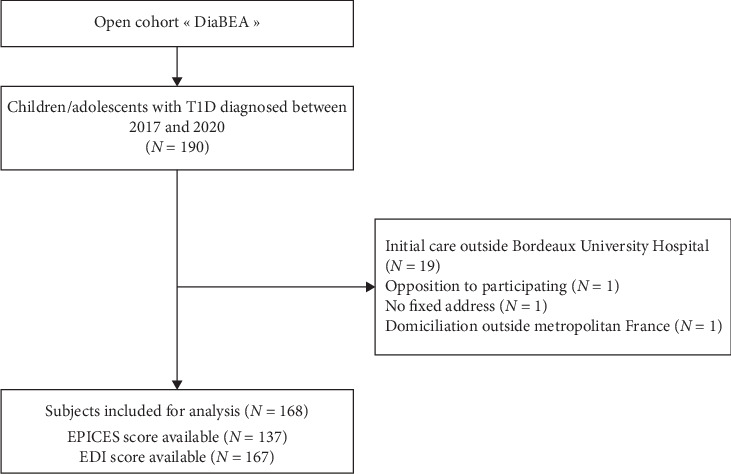
Flowchart of patient selection. DiaBEA Cohort, France, 2017–2020.

Among the 190 cases of T1D diagnosed between 2017 and 2020 in the DiaBEA center, 168 met the inclusion criteria and were included in this study (Figure 2).

### 3.1. Baseline Characteristics of the Study Population

Patient characteristics at the time of T1D diagnosis are presented in Table 1. The median HbA1c level was 11.8% (IQR 10.8–13.4%). Approximately 45% of patients were in EDI quintiles 4 and 5, and approximately 29% of patients had EPICES scores ≥ 30.17 (corresponding to those quintiles).

### 3.2. Longitudinal Data

The median follow-up duration was 2.8 years (IQR 1.8–3.7 years), and the maximum follow-up duration was 5 years. The mean HbA1c level of each patient was calculated using the follow-up data; the median was 7.4% (IQR 7.0-7.8%), the minimum was 5.6%, and the maximum was 10%. Sixty-seven patients required at least one new hospitalization after T1D diagnosis; 18 of these patients required multiple hospitalizations. Most hospitalizations (42%) were related to the installation of insulin pumps or controlled glucose monitoring systems requiring patient and family education. Uncontrolled diabetes was the cause of hospitalization in approximately 30% of cases. Nearly 10% of hospitalizations were secondary to an acute complication of T1D: ketoacidosis (3%) or severe hypoglycemia (6%). The remaining hospitalizations (19%) were for reasons not associated with T1D, such as infectious disease or surgery.

### 3.3. Comparison According to Social Deprivation Level

Although the follow-up durations, visit frequencies, and numbers of hospitalizations were similar among groups, the reasons for hospitalization differed. According to EPICES scores, patients from the least-deprived families were more often hospitalized for installation of diabetes devices (47% of all hospitalizations) or for reasons unrelated to T1D (26%), whereas patients from the most-deprived families were mostly hospitalized with uncontrolled diabetes (48%) or acute complications (19%). The EDI scores revealed that hospitalizations with acute complications were more common among patients in the most-deprived quintiles.

### 3.4. Relationship between EPICES and EDI Scores

The two scores were compared for the 136 patients who had undergone assessment of both scores. The scores were poorly correlated (Spearman correlation coefficient *r*_s_ = 0.27, *P* < 2.2e − 16). The weighted *κ* coefficient also demonstrated very low concordance (0.10; *P* = 0.06). Although 19% of patients were in the same quintiles for both indicators, 55% of patients differed by only one quintile.

### 3.5. Longitudinal Trajectories of HbA1c Levels by Social Deprivation

**Figure 3:**
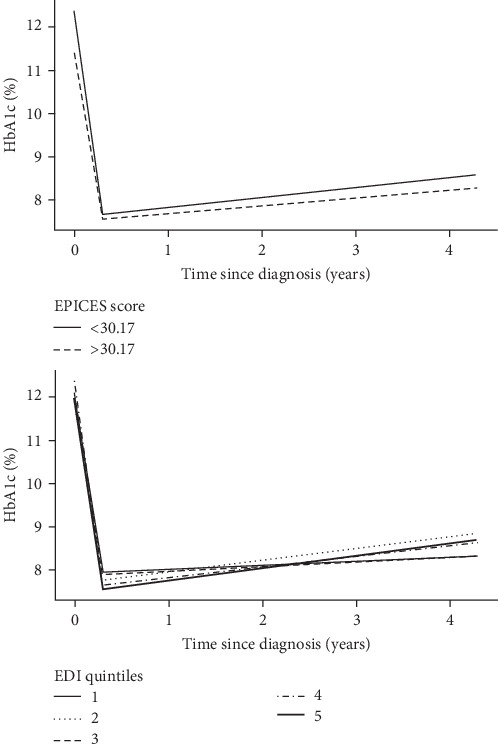
Predicted mean HbA1c trajectories according to EPICES and EDI scores derived using linear mixed models.

The results of multivariable models of changes in HbA1c level that considered indicators of social deprivation and confounders are shown in Table 2.  Figure 3 shows the predicted longitudinal trajectories of HbA1c levels according to social deprivation status. The mean HbA1c level at diagnosis was slightly lower in patients with EPICES scores ≥ 30.17, but it did not differ among patients with different extents of area-level social deprivation as measured by the EDI score. The short-term change in HbA1c slope was slightly affected by individual deprivation, as determined using the EPICES score; however, the difference was not statistically significant. The decrease in HbA1c level was smaller in the most-deprived patients over the first 4 months after diagnosis with diabetes (slope difference 2.68% per year compared with the slope among the least-deprived patients; *P* = 0.056). The EDI quintiles did not predict HbA1c changes over that period of time. Conversely, the long-term HbA1c slope was associated with the EDI score but not the EPICES score. Patients living in areas of EDI quintiles 2, 4, and 5 exhibited larger long-term increases in HbA1c levels after adjustment for confounders (slope difference of 0.2% per year for quintile 5 compared with quintile 1; overall *P* = 0.033). No dose–response relationship was apparent across EDI quintiles.

## 4. Discussion

In this cohort study performed in Bordeaux, we sought to identify an association between social deprivation and glycemic control after T1D diagnosis based on the longitudinal HbA1c trajectory. Overall, the median HbA1c level during follow-up was 7.4% (IQR 7.0-7.8%); this was lower than the levels in other industrialized countries, France overall, and the New Aquitaine region [19–21]. Among the T1D patients of the current study, the proportion of those who were most deprived was lower than the proportion in France overall. Surprisingly, the HbA1c level at diagnosis was lower in patients with EPICES scores ≥ 30.17; we had expected the opposite result or no difference. Social deprivation partially predicted HbA1c deterioration after T1D diagnosis, but the results differed according to the deprivation indicator used and the time since T1D diagnosis. The short-term HbA1c changes were affected by individual social deprivation, whereas the long-term changes were affected by area-level deprivation. In both instances, the most-deprived patients exhibited the worst HbA1c status.

Few studies have examined early glycemic control in T1D patients, as well as the impacts of social deprivation on the longitudinal trajectory of HbA1c levels in the months after T1D diagnosis. A study of longitudinal data in the United Kingdom revealed that the mean HbA1c levels in the first 6 months after T1D diagnosis differed according to ethnicity rather than an ecological indicator of social deprivation [7]. Consistent with that result, we found no association between short-term HbA1c changes and the EDI score. To our knowledge, no prior study has evaluated the impact of individual social deprivation on early glycemic control. The nonsignificant association with the EPICES score that we found is plausible; the most-deprived families may require more time to assimilate the basics of managing pediatric T1D, leading to worse glycemic control in the first months after diagnosis.

Several studies have shown that individual social deprivation is associated with longer-term glycemic control [2–5, 11]. One French study showed that the mean HbA1c level measured at follow-up visits > 1 year after T1D diagnosis was 43% higher in children/adolescents with EPICES scores ≥ 30.17, compared with children/adolescents who had lower scores (*P* < 0.001) [11]. However, we found no association between the EPICES score and the HbA1c trajectory > 4 months after diagnosis. One possible explanation is that when the diabetes team learned the EPICES score, the management strategy changed. Although the visit frequencies were similar among groups, the most-deprived patients may have received longer consultations, which involved doctors, nurse educators, and dieticians. They may also have benefited from teleconsultations or additional home assistance (e.g., nurse visits). The proportion of hospitalizations to treat unbalanced diabetes was greater among patients with higher EPICES scores (48% vs. 23% of patients with lower scores). Thus, the diabetes team may have allocated additional attention to patients with higher EPICES scores, leading to more frequent hospitalization. Alternatively, the current study may have lacked adequate statistical power, or the follow-up time may have been insufficient.

Similar to our findings, some studies showed that area-level deprivation was associated with long-term HbA1c levels [6, 8, 9, 11]. The French study cited above revealed a significant association between HbA1c levels and the EDI quintiles, but the EDI did not reflect glycemic control in a manner consistent with the EPICES score [11]. One German study demonstrated an association between HbA1c level and individual-level (but not area-level) social disadvantage [4].

The low correlations between the two scores used are consistent with findings in other studies that compared individual-level and area-level deprivation scores [22–24]. The two scores capture different aspects of social deprivation.

## 5. Conclusions

Social deprivation influenced glycemic control in young patients with diabetes who were followed-up at Bordeaux University Hospital. Patients from the least-deprived areas (EDI quintile 1 at the IRIS level) exhibited more stable long-term HbA1c levels. Further research is necessary to determine why residential areas matter and to minimize the negative effects of residing in poorer regions.

## Figures and Tables

**Table 1:** Baseline characteristics (at diagnosis) of children and adolescents who were diagnosed with T1D at Bordeaux University Hospital between 2017 and 2020 (*N* = 168).

Characteristics	Available data	*n* (%)	Median (IQR*⁣*^*∗*^)	Min–max
Sex	168			
Male		84 (50.0)		
Female		84 (50.0)		
Year of diabetes diagnosis	168			
2017		38 (22.6)		
2018		45 (25.8)		
2019		43 (25.6)		
2020		42 (25.0)		
Age at diagnosis (years)	168		9.0 (6.6–11.8)	1.0–16.4
Comorbidity	168			
Celiac disease		5 (3.0)		
Thyroid dysfunction		1 (0.6)		
Family history of T1D	168	24 (14.63)		
Family structure	167			
Parents together		142 (85.0)		
Parents separated		25 (15.0)		
Mother occupations	164			
Artisans, merchants, and company managers		7 (4.2)		
Executives and higher intellectual		36 (22.0)		
Professions		33 (20.1)		
Intermediate professions		39 (23.8)		
Employees		8 (4.9)		
Workers		41 (25.0)		
No professional activity		—		
Father occupations	160			
Farm operators		4 (2.5)		
Artisans, merchants, and company managers		21 (13.1)		
Executives and higher intellectual		34 (21.2)		
Professions		22 (13.8)		
Intermediate professions		25 (15.6)		
Employees		47 (29.4)		
Workers		7 (4.4)		
No professional activity		—		
Puberty started	168	50 (29.8)		
Body mass index (*Z*-score)^1^	151		–0.8 (–1.6 to 0.2)	–3.6 to 4.2
HbA1c level at diagnosis (%)	164		11.8 (10.8–13.4)	6.2–18
Ketoacidosis at diagnosis	168	60 (35.7)		
Length of hospital stay (days)	168		10.0 (9.0–11.0)	5–25
Insulin delivery system	168			
Insulin pen		116 (69.0)		
Insulin pump		52 (31.0)		
Use of CGM*⁣*^*∗∗*^ system	168	108 (64.3)		
Distance from home to Bordeaux	168			1.5–121
University Hospital (km)			18.0 (6.9–38.6)	
EPICES score^2^	137		14.2 (6.5–32.0)	0–79.9
<30.17		97 (70.8)		
≥30.17		40 (29.2)		
EDI score	167		–0.4 (–2.0 to 1.2)	–5.2 to 16.7
1^3^		26 (15.6)		
2^3^		31 (18.6)		
3^3^		35 (21.0)		
4^3^		39 (23.3)		
5^3^		36 (21.5)		

*⁣* ^*∗*^IQR = interquartile range, *⁣*^*∗∗*^CGM = continuous glucose monitoring. ^1^Measured on day 1 of hospitalization. ^2^EPICES scores were measured at inclusion for 36 patients and during follow-up for 101 patients (median time from T1D diagnosis for these 101 patients = 2.1 years, IQR 1.3-2.9 years). ^3^National EDI quintiles.

**Table 2:** Differences in HbA1c levels at diagnosis and the slopes of changes in HbA1c during follow-up according to indicators of social deprivation (*N* = 168).

Type of model and variable	HbA1c at T1D diagnosis (%)		Short-term^1^ HbA1c change (% per year)		Long-term^2^ HbA1c change (% per year)	
	Mean	*P*	Mean	*P*	Mean	*P*
*Model 1⁣* ^*∗*^						
Intercept/slope	12.37	**<0.001**	−16.51	**<0.001**	0.22	**< 0.001**
EPICES ≥ 30.17 (vs. EPICES < 30.17)	−0.83	**0.038**	2.68	0.056	−0.02	0.721
*Model 2⁣* ^*∗*^						
Intercept/slope	11.97	**<0.001**	−14.46	**<0.001**	0.09	0.166
EDI (vs. quintile 1)		0.707		0.595		**0.033**
Quintile 2	−0.23		−0.27		0.20	
Quintile 3	0.31		−1.35		0.01	
Quintile 4	0.38		−2.72		0.16	
Quintile 5	−0.07		−1.51		0.21	

*⁣* ^*∗*^Linear mixed-effects models adjusted for family structure, distance from home to Bordeaux University Hospital, and age at diagnosis. ^1^Short-term = 0–4 months after T1D diagnosis. ^2^Long-term = >4 months after T1D diagnosis. Bold values signify *p* < 0.05.

## Data Availability

In regards to data availability, data of the study are protected under the protection of health data regulation set by the French National Commission on Informatics and Liberty (Commission Nationale de l'Informatique et des Libertés, CNIL). The data can be available upon reasonable request after a consultation with the authors.

## Additional Points

*Strengths and Limitations*. One of the strengths of the current study is its use of a longitudinal approach. Most patients underwent 10 HbA1c measurements over time. Additionally, we used two composite indicators: one was assessed at the individual level, and the other was assessed at the regional level. Thus, we recognized that social deprivation is multidimensional. Both scores have been validated in French populations, are easy to obtain, and are reproducible; they could be used in larger studies. Limitations of this work include its single-center design, which led to lower statistical power and may hinder the generalization of the findings to regions other than Bordeaux. Notably, neither social deprivation score is optimal. The EPICES score was developed >20 years ago and may no longer appropriately assess social deprivation. Furthermore, it is based on self-reported data, and there is thus a risk of information bias; some individuals' responses may have minimized their social deprivation, leading to underestimation of the association between the EPICES score and the HbA1c trajectory. The EDI score exhibits an ecological bias (i.e., errors in the inference of individual data from aggregated data) that may have been substantial in the current study. In such instances, the EDI score was not an appropriate proxy for individual social deprivation. Confounding bias may have been present because ethnicity was not considered in the analyses (relevant data were unavailable). The EPICES score, measured at inclusion for 36 patients and during follow-up for 101 patients, could change over time; the chronic pathology may have led to greater social inequality, thereby enhancing social deprivation. A major limitation of the current study is the potential difference in follow-up according to EPICES score. The diabetes care team knew all of the scores and may have adjusted patient management accordingly. We speculate that the long-term HbA1c changes caused by individual social deprivation were “erased” by a caring team. Finally, we did not measure some variables that would be interesting to know, such as the child's psychological well-being. The cohort was also too small and only covered the first few years of diabetes detection to take into account comorbidities (only six children).

## Supplementary Materials

Supplementary Materials Appendix S1: EPICES instrument.
