# Supplementary material for: Associations of Area-Level and Parental Individual-Level Social Deprivation with Glycemic Control over Time among Children with Type 1 Diabetes in France: A Longitudinal Cohort Study
Source: Pediatr Diabetes. 2024 May 20;2024:9634867. doi: 10.1155/2024/9634867 (PMC12016843; doi:10.1155/2024/9634867)
Supplement: Supplementary Materials — Appendix S1: EPICES instrument. [file 9634867.f1.docx]

**Appendix A.** EPICES instrument.

|  | **Questions** | **Yes** | **No** |
| --- | --- | --- | --- |
| 1 | Do you ever meet with a social worker? | 10.06 | 0 |
| 2 | Do you have free health insurance? | –11.83 | 0 |
| 3 | Do you live with a partner? | –8.28 | 0 |
| 4 | Do you own your home? | –8.28 | 0 |
| 5 | Are there periods during the month when you have real financial difficulties in terms of meeting your needs (e.g., food, rent, and electricity)? | 14.80 | 0 |
| 6 | Have you exercised in the past 12 months? | –6.51 | 0 |
| 7 | Have you been to a show in the past 12 months? | –7.10 | 0 |
| 8 | Have you been on vacation in the past 12 months? | –7.10 | 0 |
| 9 | In the past 6 months, have you had contact with family members other than your parents or children? | –9.47 | 0 |
| 10 | In the event of difficulties, are there people around you that you can count on to accommodate you for a few days? | –9.47 | 0 |
| 11 | In the event of difficulties, are there people around you that you can count on for material help? | –7.10 | 0 |
|  | **Constant** | **75.14** | |

It is essential that all questions are answered. Each coefficient is added to the constant 75.14.
